# Convex–concave and anterior–posterior spinal length discrepancies in adolescent idiopathic scoliosis with major right thoracic curves versus matched controls

**DOI:** 10.1007/s43390-022-00566-w

**Published:** 2022-09-13

**Authors:** Steven de Reuver, Nick de Block, Rob C. Brink, Winnie C. W. Chu, Jack C. Y. Cheng, Moyo C. Kruyt, René M. Castelein, Tom P. C. Schlösser

**Affiliations:** 1 Department of Orthopedic Surgery, University Medical Center Utrecht, G05.228, P.O. Box 85500, 3508 GA Utrecht, The Netherlands; 2 Department of Imaging and Interventional Radiology, Prince of Wales Hospital, The Chinese University of Hong Kong, Shatin, Hong Kong; 3 Department of Orthopaedics and Traumatology, Prince of Wales Hospital, The Chinese University of Hong Kong, Shatin, Hong Kong

**Keywords:** Adolescent idiopathic scoliosis, Anterior lengthening, Scoliosis surgery, CT

## Abstract

**Purpose:**

The apical deformation in adolescent idiopathic scoliosis (AIS) is a combination of rotation, coronal deviation and passive anterior lengthening of the spine. In AIS surgery, posterior–concave lengthening or anterior–convex shortening can be part of the corrective maneuver, as determined by the individual surgeon’s technique. The magnitude of convex–concave and anterior–posterior length discrepancies, and how this needs to be modified to restore optimal spinal harmony, remains unknown.

**Methods:**

CT-scans of 80 pre-operative AIS patients with right convex primary thoracic curves were sex- and age-matched to 80 healthy controls. The spinal length parameters of the main thoracic curves were compared to corresponding levels in controls. Vertebral body endplates and posterior elements were semi-automatically segmented to determine the length of the concave and convex side of the anterior column and along the posterior pedicle screw entry points while taking the 3D-orientation of each individual vertebra into account.

**Results:**

The main thoracic curves showed anterior lengthening with a mean anterior–posterior length discrepancy of + 3 ± 6%, compared to a kyphosis of − 6 ± 3% in controls (*p* < 0.01). In AIS, the convex side was 20 ± 7% longer than concave (0 ± 1% in controls; *p* < 0.01). The anterior and posterior concavity were 7 and 22 mm shorter, respectively, while the anterior and posterior convexity were 21 and 8 mm longer compared to the controls.

**Conclusions:**

In thoracic AIS, the concave shortening is more excessive than the convex lengthening. To restore spinal harmony, the posterior concavity should be elongated while allowing for some shortening of the posterior convexity.

## Introduction

From earlier three-dimensional (3D) morphometric studies on adolescent idiopathic scoliosis (AIS) it is known that besides rotation and coronal plane deviation, the apical deformation in AIS involves lordosis, mostly by anterior opening of the intervertebral discs [1, 2]. In progressive AIS, surgical correction may be indicated. In general, scoliosis surgery aims to avoid further progression and restore ‘healthy’ spinal and trunk morphology as much as possible. Posterior scoliosis correction and spinal fusion is the most common technique used for AIS surgery, with in recent meta-analyses good long-term clinical outcomes [3–5].

There are many different ways AIS can be corrected by posterior spinal fusion [3]. In general, 3D correction maneuvers of the thoracic curve normally consist of a complex combination of concave lengthening, convex shortening and medial translation of the apex back to the midline in the coronal plane, derotation in the axial plane and posterior apical translation and thoracic kyphosis restoration in the sagittal plane. Depending on the individual surgeon’s preference, screws, hooks or laminar bands are used, and a certain strategy is chosen in which the corrections of the three main deformations (apical rotation, coronal curvature, thoracic lordosis) are prioritized, since usually not all three components can be fully reversed [3, 6–9].

That scoliosis is the complex result of deformation in all three anatomical planes is generally accepted. It is unknown, however, to what magnitude different sides of the spine lengthen or shorten in the pathogenesis of AIS. These patho-anatomical data can be helpful for further optimization of applied correction techniques in AIS surgery for 3-D restoration towards the morphology of a healthy spine. Therefore, the purpose of this study is to determine the convex–concave and anterior–posterior spinal length discrepancies of the main thoracic curve in primary thoracic AIS.

## Methods

### Study population

From existing pre-operative computed tomographic (CT) databases, patients with right-sided primary thoracic AIS (Lenke 1–4) and sex- and age matched healthy controls were included [10–14]. These patients received these CT-scans as part of their pre-operative work-up for planning of navigation guided pedicle screw placement, which in that university medical center is part of standard care for all idiopathic scoliosis patients with an indication for posterior instrumentation. Exclusion criteria were age < 10 or > 21 years, a left convex main thoracic curve, a primary lumbar curve, a main thoracic curve radiographic Cobb angle below 45° or insufficient CT-scan quality. Full-body CT-scans of the controls were acquired for indications not related to the spine, for example screening for infection, trauma or malignancy. For the included AIS patients, conventional curve characteristics were measured on free standing full-spine radiographs, according to the Scoliosis Research Society guidelines [15].

### Spinal length measurements

**Fig. 1:**
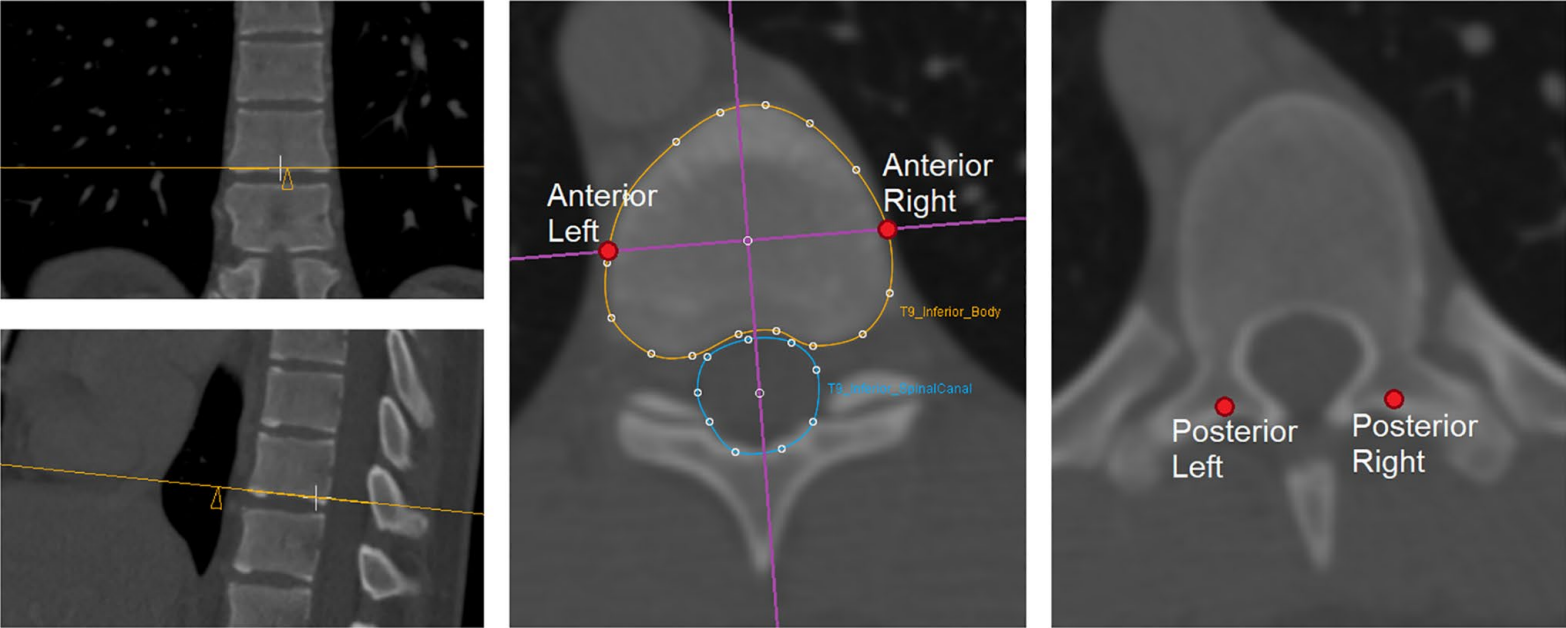
In the CT analysis software the upper and lower endplates of all vertebral bodies in the right-convex thoracic curves were segmented in the ‘true’ transverse plane. The spinal canal was also segmented to calculate the mid-sagittal axis, which intersects with the centroid of the endplate and the centroid of the canal. Perpendicular to the mid-sagittal axis at the centroid, the left and right point per was calculated. The mid-point between the upper and lower left point was calculated to retrieve the left anterior column point for each vertebrae, and similarly for the right side. Finally, on the transverse plane, where the intersections of the laminae and transverse process were visible, this was segmented to retrieve the left and right posterior column point per vertebrae

**Fig. 2:**
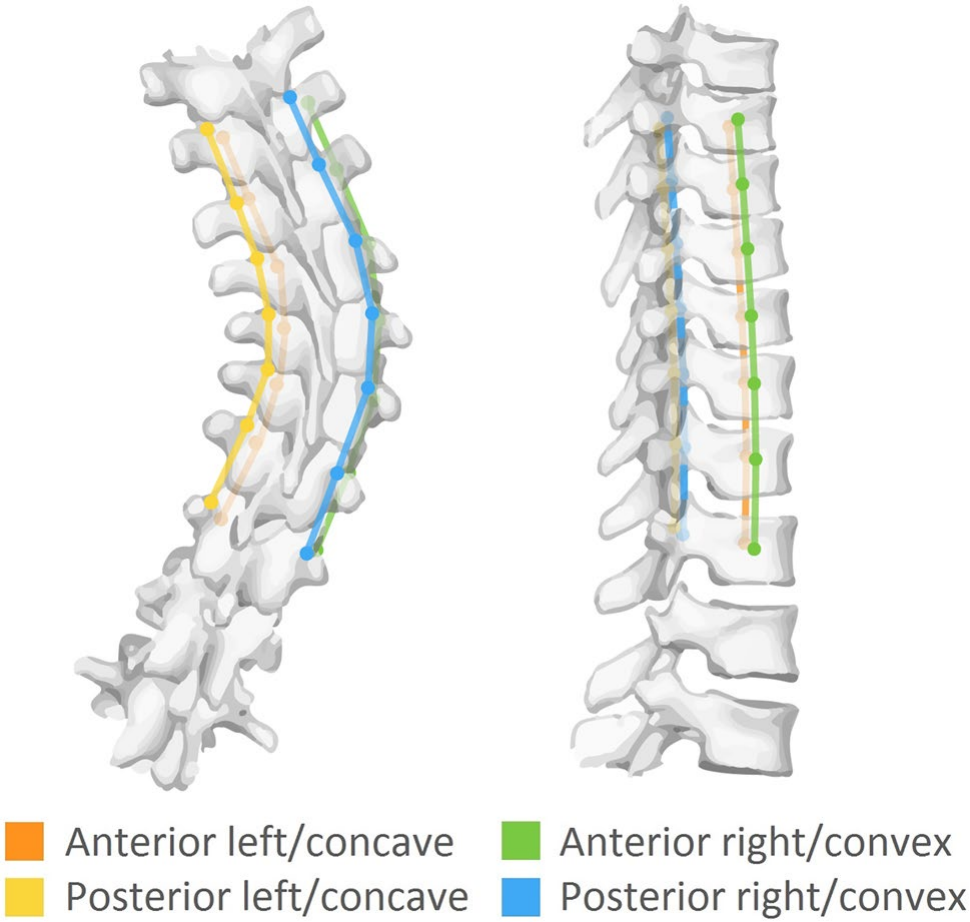
Schematic figure from a posterior and right-side view of a right-convex thoracic scoliosis and four spinal length measurements of the Cobb-end to Cobb-end segment. Following segmentation (Fig. 1) four points per vertebral body were calculated: anterior left, anterior right, posterior left and posterior right. This was done for all vertebrae in each right-convex thoracic AIS curve from Cobb-end to Cobb-end, and the same segment in a sex-age matched control. These points were connected on each side to retrieve the four spinal length measurements

For each patient, the main thoracic curve was analyzed on the CT scan from Cobb-end to Cobb-end vertebra, as determined on the standing radiographs. For the matched control, the identical levels as in the AIS patient were analyzed. The CT-scan analyses were performed using in-house developed software (*ScoliosisAnalysis 7.2, developed with MeVisLab, MeVis Medical Solutions AG, Bremen, Germany*), that was previously validated for curve morphology assessment [2, 11, 16, 17]. One observer, semi-automatically segmented all the upper and lower vertebral body endplates and the spinal canal in the ‘true’ transverse plane by correcting for the coronal and sagittal angulation of each endplate (Fig. 1). A line perpendicular to the mid-sagittal axis (based on the mid-points of the endplate and spinal canal) at the center of the endplate was used to localize the left and right side of each endplate. The midpoints between the upper and lower endplate points were automatically calculated and connected, to get the total concave and convex spinal length of the anterior column (Fig. 2). On the posterior side, the intersections of the laminae and the transverse processes were identified bilaterally, corresponding to the pedicle screw entry points, which allowed measurement of the total concave and convex posterior spinal length (Figs. 1, 2). To compare absolute and relative spinal length measurements and to correct for individual spinal size, the absolute data of the AIS patients was normalized based on the ratio of the mean total spinal segment length of the AIS group relative to the controls. The anterior–posterior length discrepancy was calculated as *[((anterior left* + *anterior right)–(posterior left* + *posterior right))/(posterior left* + *posterior right)]* × *100%.* The convex–concave length discrepancy was calculated as *[((anterior right* + *posterior right)–(anterior left* + *posterior left))/(anterior left* + *posterior left)]* × *100%.*

### Statistical analysis

Statistical analyses were performed in SPSS 26.0.0.1 for Windows (IBM, Armonk, NY, USA). Normality of distribution was tested via Q–Q plots. The difference in anterior–posterior length discrepancy and convex–concave length discrepancy between AIS and controls was analyzed with independent sample *t* tests. Differences in spinal length of each of the four sides was tested between the two groups with independent sample *t* tests. For the correlation with curve severity (Cobb angle) a non-parametric Spearman’s rho test was performed. The statistical significance level was set at 0.05.

## Results

### Study population

Out of 118 AIS patients in the CT-database, eighty patients with right-convex primary thoracic AIS were included and sex and age matched to eighty controls. Thirty-eight were excluded: four for their age, two with a left convex thoracic curve, 21 with a lumbar primary curve, seven with a Cobb angle < 45° and four with insufficient CT-scan quality. Patient and curve characteristics are shown in Table 1. Non-normalized spinal segment length was 146 mm in AIS and 160 mm in controls.

**Table 1:** Patient demographic and radiographic curve characteristics

	AIS patients (*n* = 80)	Matched controls (*n* = 80)
Mean age (± SD)	16.0 ± 2.5	15.8 ± 2.2
Range	10–21	10–21
Female sex	68 (85%)	68 (85%)
Right convex thoracic curve	80 (100%)	–
Mean cobb angle (± SD)	69.5° ± 12.6°	–
range	46–109°	–
Cobb angle group		
45–60°	15 (19%)	–
60–70°	36 (45%)	–
70–80°	12 (15%)	–
> 80°	17 (21%)	–
Lenke curve type		
Type 1	42 (53%)	–
Type 2	23 (29%)	–
Type 3	10 (13%)	–
Type 4	5 (6%)	–
Apex level		
T7	6 (8%)	–
T8	22 (28%)	–
T9	33 (41%)	–
T10	18 (23%)	–
T11	1 (1%)	–
Curve length		
5 vertebrae	4 (6%)	–
6 vertebrae	11 (14%)	–
7 vertebrae	31 (39%)	–
8 vertebrae	28 (35%)	–
9 vertebrae	4 (6%)	–
10 vertebrae	2 (3%)	–

### Spinal length measurements

The mean anterior–posterior length discrepancy in AIS patients, along the endplates and along the pedicle entry points, was + 3 ± 6%, representing apical lordosis, compared to − 6 ± 3% in controls, representing physiological thoracic kyphosis (*p* < 0.01). The mean convex–concave length discrepancy was + 20 ± 7% in AIS patients, compared to 0 ± 1% in controls (*p* < 0.01).

**Fig. 3:**
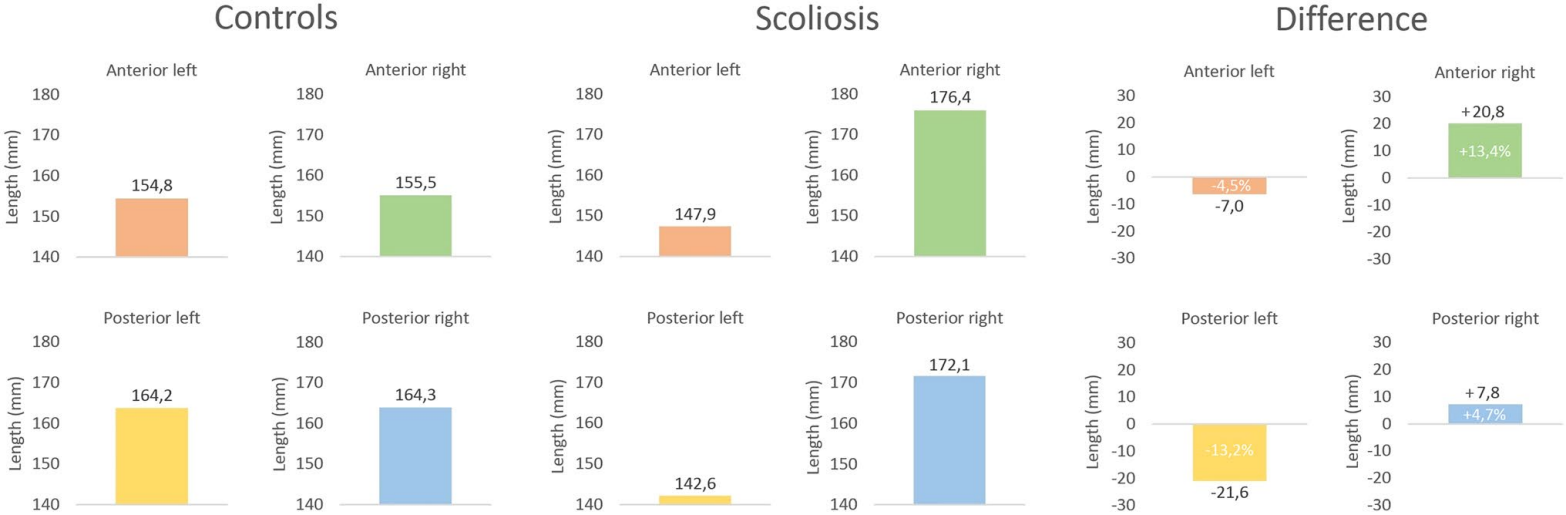
Spinal length measured over four sides in AIS patients and the same segments in sex and age matched controls. All absolute spinal length measurements of AIS patients were normalized to the length in matched controls. The mean difference between AIS and controls is also displayed

The normalized spinal length measurements (Fig. 2) along the four ‘corners’ demonstrated a mean anterior–concave length of 148 ± 29 mm in AIS versus 155 ± 30 mm in controls (difference = − 7 mm, *p* = 0.14), an anterior–convex length of 176 ± 29 mm versus 156 ± 30 mm (difference =  + 21 mm, *p* < 0.01), a posterior–concave length of 143 ± 25 mm versus 164 ± 31 mm (difference = − 22 mm, *p* < 0.01) and a posterior–convex length of 172 ± 30 mm versus 164 ± 31 mm (difference =  + 8 mm, *p* = 0.11), respectively (Fig. 3).

**Fig. 4:**
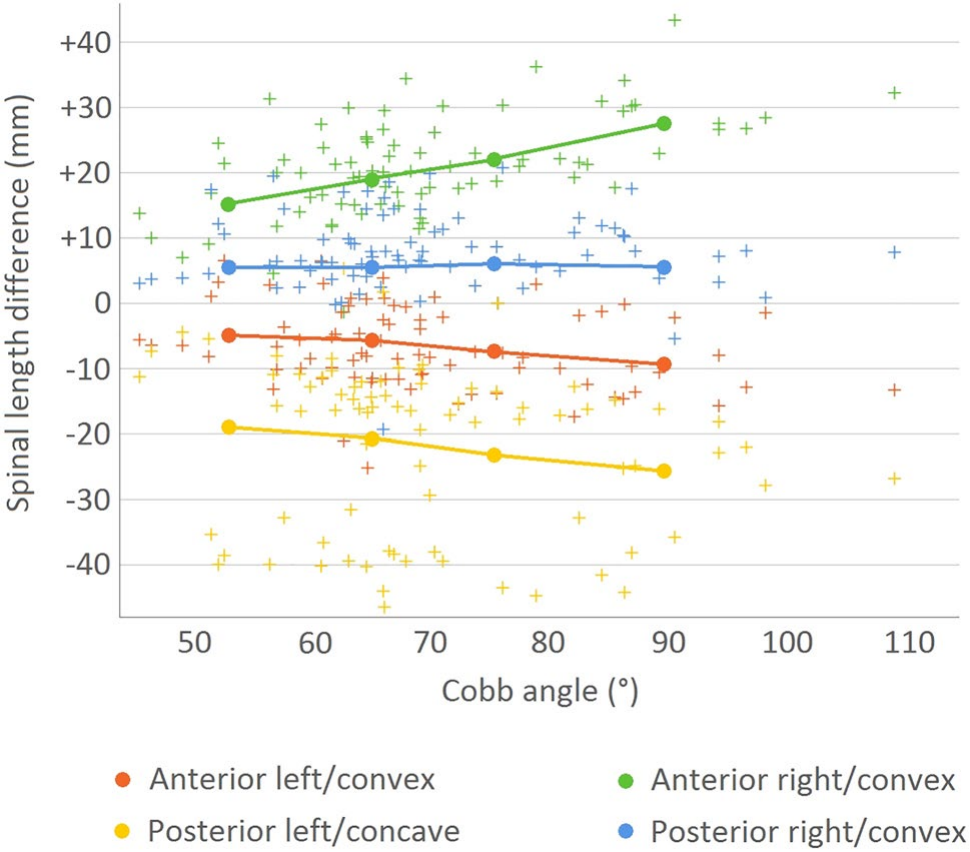
Scatter plot of the spinal length differences between AIS patients with different curve severities and the same segments in their respective sex and age matched controls. In addition, the mean values stratified per Cobb angle group: 45–60°, 60–70°, 70–80° and over 80° are overlaid

After stratifying for curve severity into four groups, Cobb angle of 45–60°, 60–70°, 70–80° and over 80° (Table 1), the length discrepancies per group were compared to their matched control as displayed in Fig. 4. The coronal curve severity Cobb angle correlated significantly with larger length discrepancies on the anterior-concavity (*p* = 0.01, *r* = − 0.29), anterior-convexity (*p* < 0.01, *r* = 0.48) and posterior-concavity (*p* = 0.01, *r* = − 0.28), but not with the posterior-convexity (*p* = 0.46).

## Discussion

In AIS surgery, much emphasis has been placed on restoring the 3D morphology of the spine to achieve a physiological alignment in all planes, to prevent adding on and junctional decompensation and to achieve an optimal configuration of the non-fused segments [3, 6–9]. Although much has been published on the shape of the scoliotic spine in the sagittal plane, no studies have so far addressed the length discrepancies along the convex and concave side of the scoliotic curvature. The present sex- and age-matched cross-sectional CT-scan analysis confirms that there is an anterior–posterior length discrepancy: a lordosis (+ 3%) is present in the true sagittal plane in thoracic AIS compared to a normal kyphosis (− 6%) in controls. This study adds to the previous literature a quantification of the convex–concave length discrepancy in AIS in the ‘true’ coronal plane: for the anterior and posterior column combined, the convexity is on average 20% longer than the concavity in AIS. When the length differences of the main thoracic curve in AIS were compared along the four ‘corners’ to the controls, the postero-concave shortening and anterior–convex lengthening were 2–3 fold greater than the contralateral lengthening/shortening, respectively (− 7 mm and − 22 mm at the anterior- and posterior-concavity, and + 21 and + 8 mm at the anterior- and posterior-convexity). With increasing curve severity, all segmental discrepancies, besides posterior–convex, increased as well.

Recent studies have already shown the anterior–posterior length discrepancy in AIS compared to controls, which is present as a relative anterior lengthening mostly of the intervertebral disc spaces, with less changes of the bone [1, 2, 11, 18]. When ignoring the obvious rotational component in AIS, this could be conceptualized as ‘spinal bending’ over a transverse axis. However, it was unknown to what extend spinal sides contribute to the deformity in AIS. The current study demonstrates that in AIS there are not only length differences in anterior–posterior direction, but that the direction of deformation is more oblique and there is mostly ‘spinal bending’ over the anterior-convexity and posterior-concavity (Fig. 3). In addition, and potentially relevant for AIS etiology, the posterior-convexity only lengthens a few millimeters and that this increase in length did not differ between curves that were more or less severe, unlike the 3 other spinal sides (Fig. 4). This may be explained by ligaments and/or interlocking facets that limit posterior lengthening. By passive decompensation the spine rotates around this fairly rigid posterior–convex tether acting as the fulcrum, allowing for posterior-concavity shortening and anterior-convexity lengthening. These observations cannot be explained adequately by the simple hypothesis of AIS as a generalized anterior bony overgrowth disorder based on the principles of Hueter–Volkmann [19–23].

In an attempt to restore healthy spinal morphology by scoliosis correction surgery, the length discrepancies should ideally be reversed. If a posterior approach is preferred, the present data suggest that the posterior concavity needs to be lengthened significantly, while the posterior convexity needs to be shortened to a much smaller extent. On average in this pre-operative population, in absolute numbers this corresponds to a 22 mm posterior–concave lengthening and an 8 mm posterior–convex shortening. Similarly, but reversed, this could be applied to an anterior approach, for which the data suggest that the largest correction should be on the convexity, with a 21 mm anterior–convex shortening, 3 times the amount of an anterior–concave lengthening (Figs. 3, 4). The effectiveness of posterior spinal releases for unilateral lengthening has not been investigated to date [24].

There are several limitations regarding this study. The first is the inevitable cross-sectional design, since it was impossible to prospectively study the change in morphology, foremost, because scoliosis patients are identified only with an already established scoliosis. Furthermore, CT scans were obtained once for preoperatively for navigation or planning purposes. In the future, 3-D reconstructions of biplanar radiographs may allow for longitudinal assessment of the convex–concave length discrepancies during scoliosis progression. In addition, the individual changes in sagittal alignment during growth could potentially influence the analysis [25–27]. To mitigate this in a cross-sectional study, all AIS patients were matched to a control patient of the same sex and age, and the exact same spinal segment per AIS and control pair was analyzed. Another potential limitation of this study is that AIS patients were from another ethnic population than the controls. To correct for individual spinal length differences, the AIS patients were normalized to the mean spinal length of the controls. A third limitation is that, scoliosis curve severity and spinal alignment is usually assessed in a free-standing position, mainly on full spine radiographs, while the CTs were obtained in a non-weightbearing position. To mitigate potential influence of body positioning, the curve severity and Cobb end vertebrae were measured on free-standing full spine radiographs obtained at the same time as the CT. We know that both coronal curve severity, as well as different sagittal parameters, are different in the same patient between standing and prone/supine, but that these do not differ significantly between prone and supine 3-D scans [28]. Regarding the individual differences in curve flexibility, i.e., the difference between standing Cobb angle versus on CT, we hypothesize that because in this study larger curves generally showed bigger spinal length differentials, the curve that would retain higher Cobb angles on CT, in other words the less flexible curves, would have a larger differential in a supine position, and could require more intraoperative correction than the more flexible ones.

## Conclusions

AIS is the complex result of rotation, coronal deviation and anterior lengthening of the spine, but to what extend each spinal side contributes and how much exactly should be reversed during scoliosis surgery has so far remained unknown. This sex- and age-matched CT-scan analysis confirms a lordosis in thoracic AIS compared to the kyphosis in controls. It demonstrates that the axis of spinal deformation is more oblique than in the coronal or sagittal plane: largely over the anterior–convex and posterior–concave side. This study provides rough targets of spinal length along the pedicle entry points to restore optimal spinal harmony during posterior scoliosis surgery: The posterior concavity should be distracted extensively (on average 22 mm) while allowing for a slight shortening (7 mm) on the convexity.
